# FDG‐PET/CT‐guided rebiopsy may find clinically unsuspicious transformation of follicular lymphoma

**DOI:** 10.1002/cam4.4924

**Published:** 2022-06-06

**Authors:** Aino Rajamäki, Hanne Kuitunen, Marc Sorigue, Salla‐Maarit Kokkonen, Outi Kuittinen, Kaisa Sunela

**Affiliations:** ^1^ Department of Oncology Hospital Nova of Central Finland Jyväskylä Finland; ^2^ Institute of Clinical Medicine, Faculty of Health Medicine University of Eastern Finland Kuopio Finland; ^3^ Department of Oncology Oulu University Hospital Oulu Finland; ^4^ Department of Hematology ICO‐Hospital Germans Trias i Pujol, Functional cytomics‐IJC Badalona Spain; ^5^ Department of Nuclear Medicine Oulu University Hospital Oulu Finland; ^6^ Medical Research Centre and Cancer and Translational Research Unit University of Oulu and Oulu University Hospital Oulu Finland; ^7^ Department of Oncology Kuopio University Hospital Kuopio Finland; ^8^ Department of Oncology, Tays Cancer Center Tampere University Hospital Tampere Finland

**Keywords:** FDG‐PET/CT, follicular lymphoma, histologic transformation, indolent lymphoma, SUVmax

## Abstract

**Aim:**

The purpose of the study was to evaluate the clinical impact of fluorodeoxyglucose‐positron emission tomography/computed tomography (FDG‐PET/CT) followed by a new biopsy from the site with maximum standardized uptake value (SUVmax) in case of high maximal SUV values, in detecting clinically unsuspected histologic transformations (HT) of follicular lymphoma (FL).

**Methods:**

This retrospective study included all the patients who had undergone FDG‐PET/CT during primary diagnosis or relapse of FL between 2010 and 2020 at Oulu University Hospital.

**Results:**

The diagnosis changed from an indolent disease to a transformed lymphoma in >10% (7/63) of the patients who underwent diagnostic FDG‐PET/CT. The HT risk associated with high SUVmax (>10) was 24% (7 of 29 performed biopsies). Four out of these seven patients with verified HT had no previous clinical suspicion of transformation.

**Conclusion:**

Our results suggest that a rebiopsy based on a high SUVmax in diagnostic FDG‐PET/CT is valuable in detecting clinically unsuspected HT of FL.

## Graphical abstract

Histologic transformation of follicular lymphoma often occurs locally in a limited number of disease sites and an indolent histology in a biopsy from one site does not exclude the presence of a high‐grade disease elsewhere. Our results suggest that a rebiopsy based on a high SUVmax in diagnostic FDG‐PET/CT is valuable in detecting HT after the diagnosis or relapse of follicular lymphoma

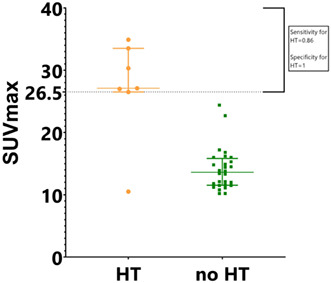

## 1 INTRODUCTION

Follicular lymphoma (FL) may undergo histologic transformation (HT) during the course of the disease. The 10‐year cumulative incidence of biopsy‐proven transformation is approximately 8%.^1^, ^2^ HT often occurs locally in a limited number of disease sites and an indolent histology from one site does not exclude the presence of a high‐grade disease elsewhere.^3^

The outcome of patients with FL has improved greatly over the last years and the 10‐year overall survival is almost 80%.^4^ However, HT is associated with increased lymphoma‐related mortality.^1^ As FL may be treated with low‐intensity regimens that are insufficient for aggressive disease, it may be speculated that differentiating cases with HT from those with purely indolent disease would have a positive impact on the survival. Among FL patients with early progression, the proportion of those patients with an undiscovered primary transformation also needs to be determined.

The aim of this study was to retrospectively evaluate the efficacy of FDG‐PET/CT followed by a new biopsy from the SUVmax area in detecting clinically unsuspected HTs.

## 2 METHODS

This retrospective study was performed in line with the principles of the Declaration of Helsinki and it was reviewed and approved by the authorities of the Northern Ostrobothnia Hospital District. All patients with a diagnosis of Grade 1 to Grade 3a FL, who had at least one visit to the oncology department in Oulu University Hospital between 2010 and 2020 were screened. Patients who had undergone diagnostic FDG‐PET/CT at diagnosis or relapse were included in the data. Principally, FDG‐PET/CT was performed for all new patients with a diagnosis of FL or its relapse, but the practice varied between clinicians and became more common during the last 5 years. Altogether, 397 patients were identified. Among these, 63 had undergone diagnostic FDG‐PET/CT and were deemed eligible for inclusion (Figure S1).

Patients fasted for 6 h prior to injection of FDG. A dose of 3.43 MBq/kg of ^18^F‐FDG was injected intravenously 60 min before PET imaging. PET/CT examination was performed using a combined PET/CT device (GE Discovery 690). The examination started with a low‐dose non‐enhanced CT scan from the level of the skull base to the upper thigh regions. СT acquisition was followed by the acquisition of PET emission images of 3 min per bed position using а matrix of 192 × 192. PET images were reconstructed using iterative algorithms and attenuation correction was routinely applied.

Whole‐body PET and CT images were loaded on three‐dimensional workstations for visual evaluation and data analysis (GE healthcare AW workstation or Hermes Hybrid Viewer). PET/CT images were reviewed by experienced radiologists. Areas of highest abnormal FDG uptake in lymphoma lesions were identified and the highest SUV (SUVmax) within a given 3D region of interest was reported.

An SUVmax of >10 was mainly considered the indication for a new biopsy and rebiopsy was performed at an accessible site with the highest SUVmax. Because of the real‐life nature of the study, three biopsies were taken also with a lower SUVmax and six patients were not re‐biopsied although their SUVmax exceeded 10 (*n* = 6) (Figure S1). A receiver operating characteristic (ROC) curve was used to determine the optimum cutoff for discriminating between HT and no HT. Statistical analyses were performed using IBM SPSS Statistics (version 27; IBM Corp.).

## 3 RESULTS

**FIGURE 1:**
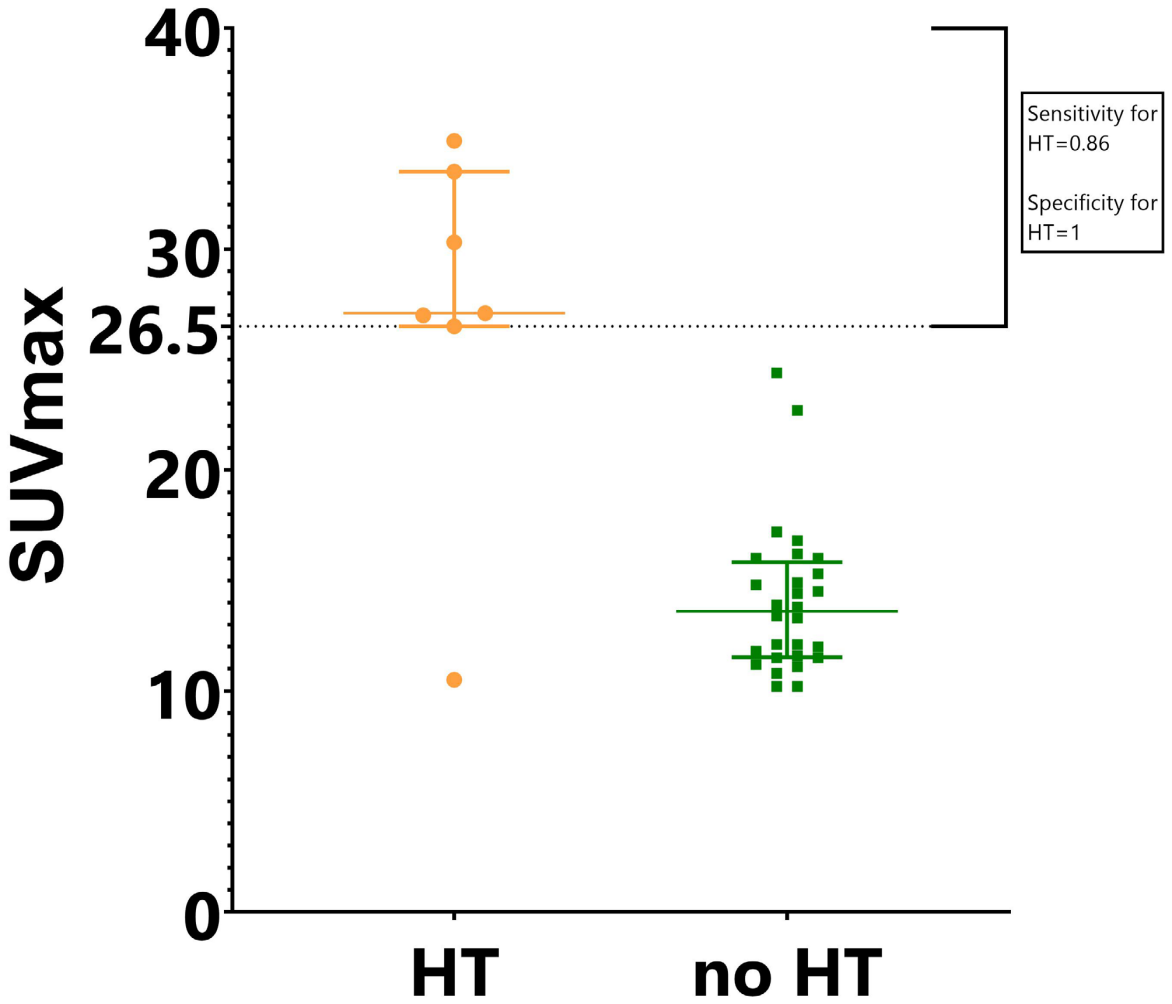
A comparison of individual SUVmax values between patients with histological transformation (HT) versus no HT, with the sensitivity and specificity for HT of the threshold 26.5. The graph includes patients with SUVmax <10 and a new biopsy (*n* = 29).

Patient characteristics are presented in Table 1. Patients were diagnosed between 1988 and 2020. The median follow‐up duration after primary diagnosis was 38 months (range: 1–382 months). Of 63 FL patients with a diagnostic FDG‐PET/CT done, a SUVmax >10 was noticed in 35 patients, and rebiopsy was taken in 29 of those. HT was found in seven out of 29 rebiopsies (24.1%), out of which six presented with diffuse large B‐cell lymphomas and one with double‐/triple‐hit lymphoma histology. The median SUVmax was higher in patients who demonstrated HT compared to those without HT but SUVmax >10 and a new biopsy (27.1 (range: 10.5–34.9) vs. 13.6 (range: 10.2–24.4), respectively) (Figure 1). The optimum SUVmax cutoff value obtained with ROC analysis was 26.5, with a sensitivity for HT of 0.86 and specificity of 1.0.

**TABLE 1:** Patient characteristics obtained at the time of the index FDG‐PET/CT performed (at the time of the possible HT)

Variable	HT (*n* = 7) *n* (%)	No HT (*n* = 56) *n* (%)
Age, years, median (range)	68 (59–88)	60 (29–87)
Sex, male	3 (42.9)	26 (46.4)
Primary diagnosis		
FL Grade 1 or 2	4 (57.1)	47 (83.9)
FL Grade 3a	3 (42.9)	9 (16.1)
Stage		
I–II	1 (16.7)	18 (32.1)
III–IV	5 (83.3)	38 (67.9)
Missing	1	0
FLIPI		
0–1	0	14 (25.9)
2	0	18 (33.3)
3–5	5 (100.0)	22 (40.7)
Missing	2	2
Bone marrow involvement		
Yes	1 (14.3)	15 (29.4)
Missing	0	5
Extranodal involvement		
Yes	3 (50.0)	15 (28.3)
Missing	1	3
LDH level		
Normal	2 (28.6)	23 (46.0)
Elevated	5 (71.4)	27 (54.0)
Missing	0	6
ECOG		
0	2 (28.6)	24 (55.8)
1	4 (57.1)	17 (39.5)
2	1 (14.3)	1 (2.3)
3	0	1 (2.3)
Missing	0	13
Hemoglobin level, g/L		
<120	2 (28.6)	6 (11.1)
≥120	5 (71.4)	48 (88.9)
Missing	0	2
B‐symptoms		
Yes	3 (42.9)	15 (28.3)
Missing	0	3
The point when FDG‐PET/CT was made		
Diagnosis	2 (28.6)	33 (60.0)
First relapse	4 (57.1)	16 (29.1)
Second relapse or later	1 (14.3)	6 (10.9)
Missing	0	1
Treatment following FDG‐PET/CT		
Anthracycline containing	6 (85.7)	16 (28.6)
Bendamustine	0	17 (30.4)
Rituximab monotherapy	0	3 (5.4)
Radiotherapy only	0	5 (8.9)
Watchful waiting	0	13 (23.2)
Other^a^	1 (14.3)	2 (3.6)

Abbreviations: B‐symptoms, systemic symptoms (unexplained weight loss, fever, night sweats); ECOG, Eastern Cooperative Oncology Group performance status; FDG‐PET/CT, fluorodeoxyglucose‐positron emission tomography/computed tomography; FLIPI, Follicular Lymphoma International Prognostic Index; LDH, lactate dehydrogenase.

^a^ Other treatments included R‐DHAP (rituximab, dexamethasone, cytarabine, and cisplatin) in the group of HT patients, and O‐MINE (ofatumumab, mesna, ifosfamide, mitoxantrone, etoposide), and lenalidomide (1 each) in the group of patients with no HT.

The median time from diagnosis to transformation was 25 months (range: 0–183 months). Two HTs (28.6%) were discovered at diagnosis, four (57.1%) at first relapse, and one (14.3%) at second relapse. Over half of the new biopsies with HT (4/7) were obtained from the abdominal lymph nodes. Other sites (one each) included the cervical, supraclavicular, and axillary lymph nodes. Of the non‐HT biopsies, 24.0% were obtained from the abdominal, 16.0% from the cervical, and 12.0% from the axillary lymph nodes. The remaining biopsies were obtained from the thoracic, inguinal, and supraclavicular lymph nodes, bone, kidney, and miscellaneous sites.

Among seven patients with proven HT, in four patients there was no previous clinical suspicion of transformation (one PET image is shown as Figure S2). HT was clinically suspected in two patients, but the first biopsy showed indolent histology and the diagnosis of HT was confirmed on rebiopsy after FDG‐PET/CT. In one patient, HT was clinically suspected and the biopsy was obtained directly based on FDG‐PET/CT.

The median follow‐up after FDG‐PET/CT was 19 months (range: 0–97 months). During surveillance three other patients experienced HT. One HT was diagnosed within 6 months of the FDG‐PET/CT and two were diagnosed >1 year after the investigation. Median SUVmax of these patients in previous FDG‐PET/CT was 9.0 (range: 8.9–9.8). There were six patients who were not re‐biopsied although their SUVmax exceeded 10. In one patient, the initial biopsy was taken from the SUVmax area and the biopsy was not repeated. In two patients, lymphoma was treated as a transformed disease without a histological verification. One patient was taking part in a clinical study, and in two patients the reason was not found in medical records retrospectively. During the follow‐up, no HTs were histologically confirmed among these patients.

Fifty out of 63 patients received treatment after FDG‐PET/CT (Table 1). Among these, 76.0% received immunochemotherapy, 6.0% received rituximab monotherapy, and one patient (2.0%) received maintenance rituximab. Nine patients showed disease progression during follow‐up after FDG‐PET/CT. The 5‐year progression‐free survival (PFS) was 37.9% and the median PFS was 52 months. There were no lymphoma‐related deaths, while two deaths were due to other reasons.

## 4 DISCUSSION

In this retrospective study of 63 patients with FL, a rebiopsy based on a high SUVmax in FDG‐PET/CT was valuable in detecting HT. As HT is coupled with poor prognosis, revealing patients with HT at an early stage is essential.

Very few studies have evaluated the role of FDG‐PET/CT in detecting clinically unsuspected transformations in patients with FL. Noy et al,^5^ and Karam et al^6^ discovered a higher SUVmax in transformed tumors than in non‐transformed tumors. However, a recent study by Mir et al^7^ including 549 patients reported that neither the intensity of the FDG uptake (SUVmax) nor the variation in the uptake level (SUVrange) of de novo FL patients predicted subsequent HT. These findings suggest that there might be only a minor benefit in performing a rebiopsy to exclude HT based on SUVmax alone before initiating the therapy. However, in that study, patients with biopsy‐confirmed HT based on PET findings at baseline were not included. The median time between baseline PET scan and biopsy‐confirmed HT was 11.1 months. Most probably, transformations were not present at baseline. All patients were treated with immunochemotherapy, which might have treated a proportion of undetected transformations.

HT is often suspected based on clinical presentation. Factors associated with an increased risk of transformation include age older than 60 years, elevated lactate dehydrogenase, high Follicular Lymphoma International Prognostic Index scores, B‐symptoms, an advanced disease, more than one extranodal site, poor Eastern Cooperative Oncology Group performance status (>1), tabsence of response to frontline therapy, and Grade 3a FL. The use of rituximab reduced the risk of HT.^1^, ^2^, ^7^, ^8^ Similar findings were also seen in our material (Table 1). Moreover, features associated with clinical suspicion of HT include rapid growth of tumor masses, the rapid development of symptoms, and/or clinical deterioration or change in the tempo of a previously indolent disease course.^3^ Importantly, four out of seven patients had no previous clinical suspicion of transformation in our study.

The present study has some limitations. The study population was small with only seven observed HTs, leaving room for a possibility of bias. The median follow‐up was short so the potential survival advantage of an FDG‐PET/CT cannot be analyzed. New biopsies were mainly obtained only from the patients whose SUVmax exceeded 10. Hence, we cannot exclude the possibility of some HTs with lower SUVmax values. This protocol was based on the existing literature suggesting that a high SUVmax (>10) on FDG‐PET/CT predicts aggressive lymphoma and therefore, could also predict transformation.^9^, ^10^ Indeed, the optimum cutoff value obtained with ROC analysis was surprisingly high, 26.5. However, considering the limited number of patients included in this study, we speculate that a portion of HTs might not be detected with these high SUVmax cutoffs. Among the 29 patients with a SUVmax >10 and a new biopsy, 24.1% were diagnosed with HT, suggesting that in cases where SUVmax exceeds 10, a rebiopsy from the area with the highest SUVmax might be beneficial.

The encouraging preliminary results of the benefits of diagnostic FDG‐PET/CT followed by a new biopsy from the SUVmax area in detecting HT call for further research with larger populations and longer follow‐up periods. This would enable the analysis of the potential survival advantage offered by diagnostic FDG‐PET/CT and rebiopsy.

## AUTHOR CONTRIBUTIONS

Aino Rajamäki, Outi Kuittinen, and Hanne Kuitunen designed the research. Aino Rajamäki collected the data. Aino Rajamäki, Salla‐Maarit Kokkonen, Outi Kuittinen, and Kaisa Sunela analyzed the results and wrote the original draft. Aino Rajamäki and Marc Sorigue made the figures. Hanne Kuitunen and Marc Sorigue reviewed and edited the manuscript. Outi Kuittinen and Kaisa Sunela supervised the study. All the authors accepted the final version of the manuscript.

## FUNDING INFORMATION

This work was funded by the Finnish Blood Disease Research Foundation and Northern‐Savonia Cancer Society (A.R.). Other authors disclose no special funding for this research.

## CONFLICT OF INTEREST

The authors have no relevant financial or non‐financial interests to disclose.

## ETHICAL APPROVAL AND PATIENT CONSENT

No consent from the Ethical Board or patients was required for the study, as Finnish and EU legislation allows the use of register‐based data for retrospective studies without the informed consent of the patients concerned. Permission to use patient‐related data was applied from the hospital authorities responsible for that data.

## Supporting information


Figure S1

Figure S2

## Data Availability

The datasets generated during the current study are available from the corresponding author for reasonable requests.
