# Supplementary material for: FDG‐PET/CT‐guided rebiopsy may find clinically unsuspicious transformation of follicular lymphoma
Source: Cancer Med. 2022 Jun 6;12(1):407–11. doi: 10.1002/cam4.4924 (PMC9844644; doi:10.1002/cam4.4924)
Supplement: Supplementary file 1 — Figure S1 [file CAM4-12-407-s001.docx]

Patients identified

N = 397

No diagnostic FDG-PET/CT

Missing information of PET scan results or pathology report

Children under 18 years

Underwent a diagnostic FDG-PET/CT

N = 63

SUVmax > 10

N = 35

SUVmax < 10

N = 28

Patients with no new biopsy:

2 were considered transformations and treated accordingly without a new biopsy

1 patient had first biopsy taken from SUVmax area already

1 patient was taking part in a clinical study

2 patients with no information of the reason

Patients with new biopsy:

1 patient with suspicion of another malignancy because of the FDG-PET/CT

1 biopsy to confirm the relapse (no previous biopsy)

1 biopsy to confirm the diagnosis of cutaneous FL

New biopsy

N = 3

New biopsy

N = 29

HT detected by PET scan

N = 7

HT detected by PET scan

N = 0
