# Supplementary figures and images for: FDG‐PET/CT‐guided rebiopsy may find clinically unsuspicious transformation of follicular lymphoma
Source: Cancer Med. 2022 Jun 6;12(1):407–11. doi: 10.1002/cam4.4924 (PMC9844644; doi:10.1002/cam4.4924)

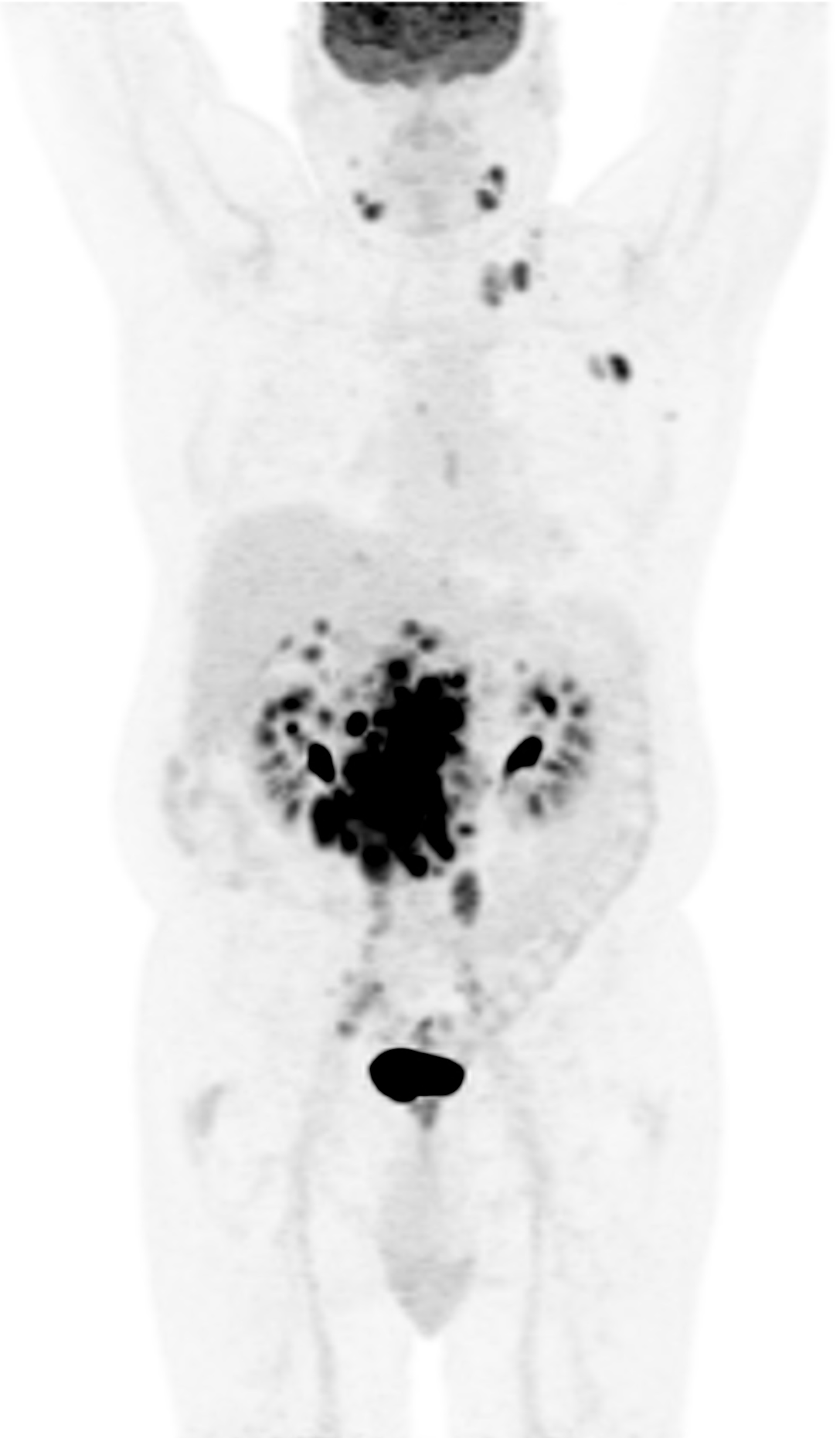

Supplement: Supplementary file 2 — Figure S2 [file CAM4-12-407-s002.png]
